# Cobalt Phthalocyanine Modified Electrodes Utilised in Electroanalysis: Nano-Structured Modified Electrodes *vs.* Bulk Modified Screen-Printed Electrodes

**DOI:** 10.3390/s141121905

**Published:** 2014-11-19

**Authors:** Christopher W. Foster, Jeseelan Pillay, Jonathan P. Metters, Craig E. Banks

**Affiliations:** 1 Faculty of Science and Engineering, School of Chemistry and the Environment, Division of Chemistry and Environmental Science, Manchester Metropolitan University, Chester Street, Manchester M15 GD, Lancs, UK; E-Mails: cwfoster90@gmail.com (C.W.F.); jpmetters@gmail.com (J.P.M.); 2 Nanotechnology Innovation Centre, Advanced Materials Division, Mintek, 200 Malibongwe Drive, Randburg 2125, South Africa; E-Mail: jessiep@mintek.co.za

**Keywords:** cobalt nanophthalocyanine, cobalt phthalocyanine screen-printed electrodes, electrocatalysis, sensing

## Abstract

Cobalt phthalocyanine (CoPC) compounds have been reported to provide electrocatalytic performances towards a substantial number of analytes. In these configurations, electrodes are typically constructed *via* drop casting the CoPC onto a supporting electrode substrate, while in other cases the CoPC complex is incorporated within the ink of a screen-printed sensor, providing a one-shot economical and disposable electrode configuration. In this paper we critically compare CoPC modified electrodes prepared by drop casting CoPC nanoparticles (nano-CoPC) onto a range of carbon based electrode substrates with that of CoPC bulk modified screen-printed electrodes in the sensing of the model analytes l-ascorbic acid, oxygen and hydrazine. It is found that no “electrocatalysis” is observed towards l-ascorbic acid using either of these CoPC modified electrode configurations and that the bare underlying carbon electrode is the origin of the obtained voltammetric signal, which gives rise to useful electroanalytical signatures, providing new insights into literature reports where “electrocatalysis” has been reported with no clear control experiments undertaken. On the other hand true electrocatalysis is observed towards hydrazine, where no such voltammetric features are witnessed on the bare underlying electrode substrate.

## 1. Introduction

The importance of electrocatalysis continues to be a major interest to chemists and engineers since the ability to provide a “clean” system which does not contaminate or foul the electrode surface is vital in a range of applications such as electroanalytical sensors, corrosion chemistry and energy conversion devices (*i.e.*, hydrogen fuel cell and batteries) to name just a few [1,2]. Chemical moieties have been extensively used, as well as transition metals such as iron, cobalt and copper phthalocyanines that represent a significant focal point of research [3–5]. These macrocyclic compounds have been reported to exhibit electrocatalytic responses compared to the underlying (bare) supporting electrode substrate. For example, the sensing of hydrogen peroxide has been extensively studied using metal phthalocyanines [6–8], and elegant work by Ozoemena *et al.* [9] has shown that a cobalt phthalocyanine (CoPC)-cobalt (II) tetraphenylporphyrin (CoTPP)-glucose oxidase-Nafion^®^ layer modified glassy carbon can be usefully utilised for the sensing of glucose. Additionally similar work by Kondo *et al.* [10] have reported a similar method utilising a boron-doped diamond electrode as the underlying electrode. Note that in both cases, electrocatalysis (*via* the modified CoPC electrodes) towards the sensing of hydrogen peroxide produced from the enzymatic reaction is reported compared to the bare underlying/supporting electrode substrate [9,10]. Other studies by Wring *et al.* [11] have reported CoPC to be electrocatalytic towards the analytes coenzyme A and reduced glutathione. CoPC has also been used extensively towards the sensing of hydrazine where in many cases the literature highlights its excellent electrocatalytic properties [11–14], while additionally other toxic nitrogenous compounds such as aziprotryne [15] and amitrole [16] have been targeted.

**Scheme 1.**
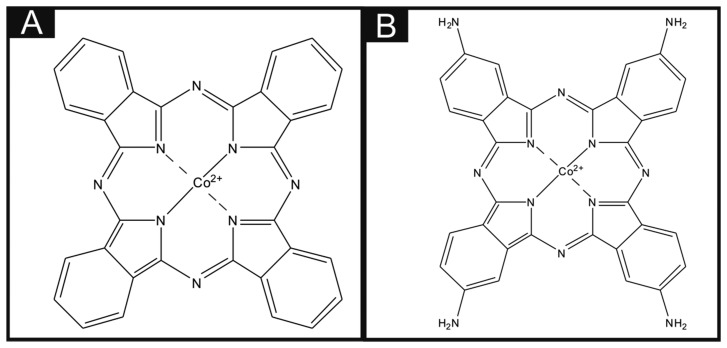
Molecular structures of the CoPC complexes used in this study, (**A**) shows CoPC structure incorporated within the ink of the screen-printed electrodes (bulk-CoPC SPEs); while (**B**) shows the nano-CoTAPC with secondary amine groups bonded at each benzene ring of the conjugated system.

Throughout the literature it is apparent that there are differences in the utilisation of metal phthalocyanines as an electrocatalytic material. There is a vast amount of literature concerning the drop casting technique of a dispersion of phthalocyanines (for example, CoPC within a suitable solvent) onto the surface of carbon-based electrodes, and this technique has been utilised towards the sensing of many analytes with much success. For example, Caro *et al.* [17] studied the electrocatalytic effect of CoPC in the sensing of nitrite using a CoPC-modified vitreous carbon electrode. Other studies by Matemadombo *et al.* [18] have reported the sensing of l-ascorbic acid and have compared the use of surface-modified graphitic screen-printed electrodes and a rotating disk electrode, with significant findings in favour for the use of screen-printed electrodes with electrocatalysis reported utilising the CoPC. Work by Wang *et al.* [19] explored the effect of nano-CoPC (shown in Scheme 1A) towards l-ascorbic acid as an ionophore, with promising electrocatalytic effects compared to a bare glassy carbon electrode. Similar studies by Agboola *et al.* [20] and Pillay *et al.* [21] have examined the effect of nano-CoPC towards analytes such as epinephrine, dopamine and ascorbic acid, where in all instances improved electrochemical responses when combined with single-wall carbon nanotubes supported upon an edge plane pyrolytic graphite electrodes (EPPGE) have been demonstrated [20,21]. Such approaches are reported to encompass numerous benefits such as alterations in mass transport, a large specific surface area, high selectivity and control over microelectrode environment [22]. Other work has reported bulk sulfonated-CoPC in a polypyrrole matrix for ammonia gas sensing [23], multi-walled carbon nanotubes-cobalt phthalocyanine (MWCNTs-CoPC) nanocomposites [24] and a graphene oxide-CoPC hybrid material as a new electrocatalyst for the electrooxidation of l-cysteine, to name just a few [25].

In addition to the above approaches where bulk and CoPC nanoparticles are drop casted onto the desired electrode surface, an alternative is the use of bulk-CoPC screen-printed electrodes (bulk-CoPC SPEs) where the CoPC is incorporated into the ink used to fabricate the screen-printed electrodes allowing the mass production of reproducible CoPC modified screen-printed electrodes. Such electrodes have been explored towards the sensing of model analytes such as citric acid and hydrazine, resulting in an electrocatalytic response when utilising the CoPC modified electrodes compared to graphitic SPEs and give rise to highly reproducible, one-shot economical and disposable electrode configurations [26,27].

To the best of our knowledge, there has been no direct comparison of drop casting nano-CoTAPC (shown in Scheme 1B) and CoPC powder upon electrode surfaces with that of using bulk-CoPC screen-printed electrodes. Consequently in this paper, we make this critical comparison towards the model analytes l-ascorbic acid, oxygen and hydrazine. Interestingly, we find that in the case of the electrochemical oxidation of ascorbic acid that the widely reported electrocatalysis of CoPC was not observed and that the bare underlying/supporting carbon electrode provides a useful electroanalytical response. The electrocatalysis of oxygen by CoPC is found to occur to an extent compared to the bare underlying electrode while extensive electrocatalysis is observed for the electrochemical oxidation of hydrazine. Such work is of importance for those considering the use of CoPC and which electrode modification might best suit the needand intended application.

## 2. Experimental Section

All chemicals used were of analytical grade and were used as received without any further purification and were obtained from Sigma-Aldrich (St. Louis, MO, USA). All solutions were prepared with deionised water of resistivity not less than 18.2 MΩ cm. Voltammetric measurements were carried out using a Palmsens Emstat (Palmsens, Utrecht, Netherlands) potentiostat.

Experiments carried out throughout this study consisted of a three electrode system, using graphitic screen-printed electrodes (standard-SPE), cobalt (II) phthalocyanine nanoparticle modified screen-printed electrodes (nano-CoTAPC SPE) and cobalt (II) phthalocyanine screen-printed electrodes (bulk-CoPC SPE) as the defined working electrodes, with a nickel counter and a saturated calomel electrode (SCE) as the reference electrode completing the circuit.

The standard-SPEs were fabricated in-house with appropriate stencil designs to achieve a 3 mm diameter working electrode, using a microDEK 1760RS screen-printing machine (DEK, Weymouth, UK). A carbon-graphite ink formulation (Product Code: C2000802P2; Gwent Electronic Materials Ltd., Pontypool, UK) was next printed onto the polyester (250 μm thickness, Autostat™, Oxford, UK). This layer was cured in a fan oven at 60 degrees Celsius for 30 min. Finally, a dielectric paste (Product Code: D2070423D5; Gwent Electronic Materials Ltd.) was then printed onto the polyester substrate to cover the connections. After curing at 60 degrees Celsius for 30 min the screen-printed electrodes are ready to be used. Note that this work was conducted with an SCE reference electrode, however the screen-printing of a reference electrode utilising a silver/silver chloride ink is feasible if required, for example as would be necessary for application “into-the-field”. For the bulk-CoPC SPEs a carbon-graphite ink formulation, with the mediator CoPC (Product code: C2030408P3; Gwent Electronic Materials Ltd.) was used throughout, with a molecular structure shown in Scheme 1A. The same printing method described above was also used to fabricate these SPEs. A glassy carbon electrode (GCE) (3 mm diameter, BAS, West Lafayette, IN, USA) and a boron-doped diamond electrode (BDDE) (3 mm diameter, BAS, West Lafayette, IN, USA) were also utilised. The BDDE and GCE were both thoroughly cleaned and polished with 1 μm and 0.25 μm diamond sprays before use.

**Figure 1.**
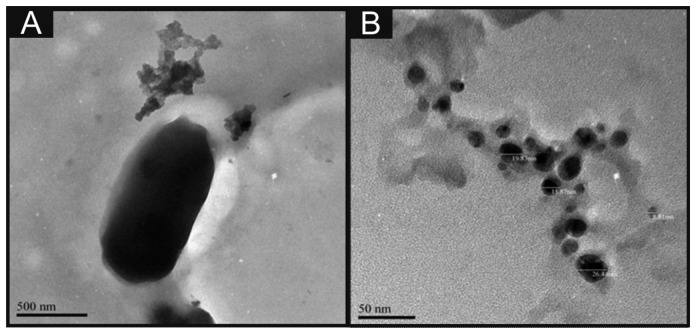
TEM images of the CoTAPC prior to the reaction with (CTACl; C1_6_H_33_N(CH_3_)_3_Cl)-CTAB (**A**) and the nano-CoTAPC (**B**) as a result of this reaction with the sizes ranging from 8.81 nm to 26.4 nm.

The CoPC nanoparticles (termed nano-CoTAPC herein) have a slightly different molecular structure than the CoPC used in the bulk-CoPC SPEs (as shown in Scheme 1B). The nano-CoTAPC were synthesized as described previously [28] with a slight modification. Briefly, CoTAPC (0.15 g, shown in Figure 1A) was dissolved in 98% concentrated sulfuric acid (5 mL). The solution was then added drop-by-drop into a vigorously stirred aqueous solution (300 mL) containing hexadecyltrimethyl ammonium-chloride ((CTACl; C1_6_H_33_N(CH_3_)_3_Cl)-CTAB, 0.45 g). The resulting solution was centrifugally separated. The obtained sedimentation was washed repeatedly to neutralise with water. It was then dried in air to obtain the nano-CoTAPC powder. The above mentioned working electrodes were modified with nano-CoTAPC which had been dispersed into a solvent—water mixture of ethanol–water (50:50) at an amount of 0.5 mg/mL and gently sonicated before use. The aliquots (μL) were then pipetted onto the desired electrode surface and then the electrode was placed into an oven to evaporate the solvent mixture at 40 °C for 2 min. Note that this method was compared to air/room drying at room temperature, where in the case of room temperature drying the modification tended to disperse to the edge of the working electrode; additionally this method took longer for the evaporation to take place. Consequently this drying method was utilised as such effects were not obtained. Additionally the use of CoPC powder from Sigma-Aldrich was also used to modify the standard-SPEs, using the method described previously.

Scanning electron microscope (SEM) images and surface element analysis were obtained with a JEOL JSM-5600LV (JEOL, Tokyo, Japan) model having an energy-dispersive X-ray microanalysis package. For the high resolution transmission electron microscope images a JEOL JEM 2100F was used. Figure 1B depicts a typical TEM image of the nano-CoTAPC utilised throughout this work, it is apparent that the nanoparticles have an average size of 23 nm. Zeta potential analysis of the nano-CoTAPC was found to exhibit a value of −5.37 mV with a conductivity of 0.004 mS/cm. Raman analysis was carried out using the Thermo Scientific™ DXR Raman (Themo Scientific™, Waltham, MA, USA).

## 3. Results and Discussion

**Figure 2.**
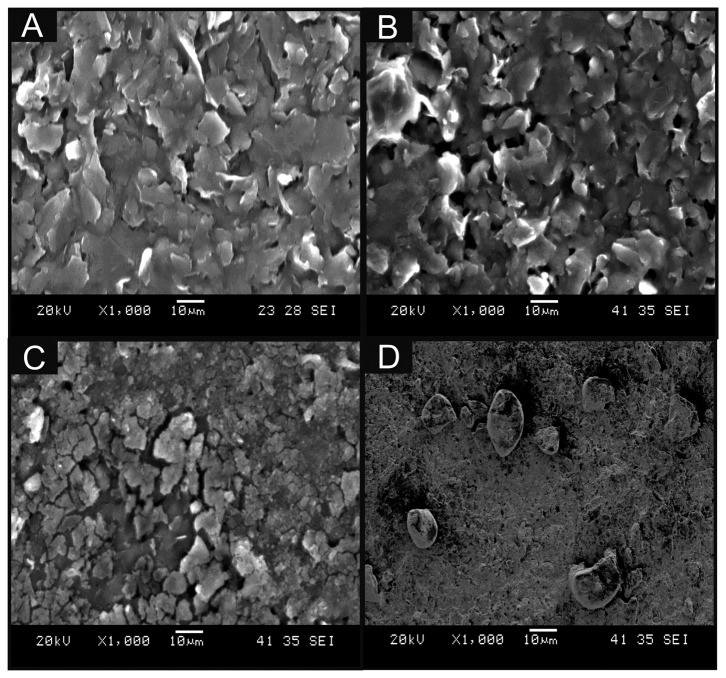
Typical SEM images of a bare standard-SPE (**A**), bulk-CoPC SPE (**B**) and a standard-SPE modified with 20 μg and 70 μg nano-CoTAPC (**C** & **D** respectively).

In this work, we critically explore bulk-cobalt (II) phthalocyanine modified screen-printed electrodes (bulk-CoPC SPE) and drop casted CoPC nanoparticles modified screen-printed electrodes (nano-CoTAPC SPE). Figure 2A displays a typical SEM image of a bare unmodified standard-SPE where the electrode surface is free of any CoPC and is in agreement with our prior work [26]. Figure 2B shows a typical SEM image of bulk CoPC-SPE where in comparison to the bare SPE (Figure 2A) there appears to be no significant morphological differences. Figure 2C,D show typical SEM images of a nano-CoTAPC modified screen-printed electrode where the CoPC nanoparticles (20 and 70 μg, respectively) have been drop cast onto the electrode surface, it is apparent that at high masses of nano-CoTAPC we witness large areas of clumping, creating a non-uniform surface. Further SEM images are presented within Figure S1 where we show the extent of using a nano-CoTAPC mass of 5 × 10^−3^ μg and increased masses of 10 μg and 30 μg, again it is clear that we create a non-uniform surface, compared to that of the bare-SPEs used throughout. Therefore the surface has led to a heterogeneous surface with an uneven coverage of nano-CoTAPC which has resulted in areas of both excessively rich and dilapidated levels of CoPC—in effect the nano-CoTAPC has coalesced on the electrode surface to form larger micron sized CoPC particles. This observation (Figure 2C) in surface morphology is in contrast to that of the bulk-CoPC SPE (Figure 2B); such observations have been similarly reported by Kozub *et al.* [5] using CoPc drop cast modified edge plane and basal plane pyrolytic graphitic electrodes. As the nano-CoTAPC SPEs are experimentally tailored with differing amounts of nano-CoTAPC, each modified electrode surface will have a different CoPC coverage. Raman analysis of the nano-CoTAPC and bulk CoPC-SPEs are presented in Figure S2, thus elaborating the CoPC coverage upon each electrode. It is clear that at very low masses of nano-CoTAPC (5 × 10^−3^ μg) the response of the chosen underlying electrode surface is favoured within this analysis. However, at larger masses of nano-CoTAPC (20 μg) it is clear that compared to the bulk CoPC-SPE a similar response is witnessed. The coverage of the electrode surface can be calculated using Equation (1):

(1) Γ=Q/nFA

where Γ, is the coverage of CoPC immobilised upon the desired electrode surface, *Q,* is the charge taken from the integration of the oxidation wave resulting from the Co ^2+/3+^ couple recorded in a pH 7.4 phosphate buffer solution (PBS) at slow scan rates, *n* is the number of electrons taking place in the electrochemical process, *F* is the Faraday constant and *A*, is the geometrical electrode area (without recourse to any surface roughness corrections). Through the use of Equation (1), a CoPC coverage value was found to correspond to 3.39 × 10^−14^ mol cm^−2^ for the bulk-CoPC SPEs while for the nano-CoTAPC SPEs, values between 1.16 × 10^−11^ to 5.80 × 10^−15^ mol cm^−2^ were obtained for immobilised CoPC masses of 5 × 10^−4^ and 7 × 10^1^ μg, respectively. Note that the CoPC in the CoPC SPEs cannot be easily changed and a new ink formulation would need to be developed by the ink supplier. Given that CoPC is a square planar molecule with a size of *ca.* 1.2 nm × 1.2 nm [29], it is possible to estimate that 1 cm^2^ of monolayer CoPC (on an ideally flat surface) should comprise a coverage of 1.2 × 10^−10^ mol cm^−2^ CoPC molecules. In comparison of this theoretical value to that of our deduced coverage values, the latter are ∼10 times smaller for nano-CoTAPC SPEs. However as shown in Figure 2, sub-monolayers of CoPC are not observed, but rather microcrystalline structures. This will affect the electrical communication with the underlying graphitic electrode surfaces since these are comprised of edge plane and basal plane sites where the former are electrochemically active “microbands” and the latter are electrochemically inert. As such, only CoPC crystals located on top of edge plane defects contribute to the electrochemical current, consequently not all of the immobilised nano-CoTAPC will be electrically wired. In terms of the bulk modified CoPC, only the surface layer is accessible to the solution and hence the rest of the electrode containing the bulk of the incorporated CoPC is likely not wired electrically. In this configuration CoPC reduces the percolation pathways depending upon its conductivity and might be detrimental to the electrochemical performance. As such, this likely explains the discrepancies observed in the deduced coverages. Additionally in comparison to that of the drop casted nano-CoTAPC electrode, the bulk-CoPC SPEs have a fixed surface CoPC distribution which cannot be easily altered and this might be a potential disadvantage.

**Figure 3.**
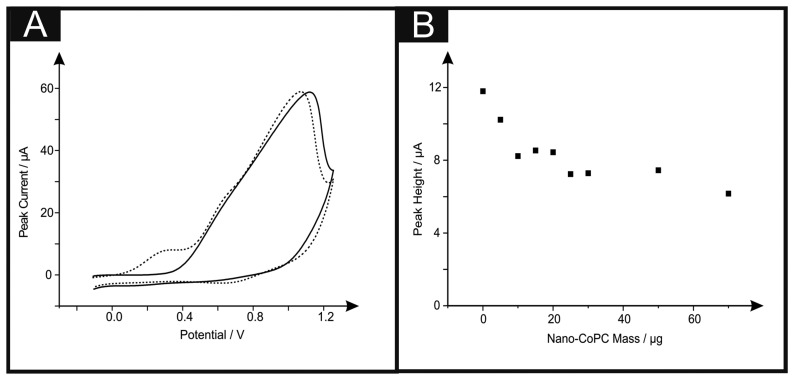
Cyclic voltammograms (**A**) in the presence (dashed line) and absence (solid line) of 1 mM l-ascorbic acid in pH 7.4 PBS utilising a standard-SPE modified with 20 μg nano-CoTAPC; (**B**) shows the corresponding plots of peak height (using the peak observed at ∼ +0.30 V *vs.* SCE) as a function of varying amounts (mass) of nano-CoTAPC. Scan rate: 100 mV s^−1^.

Attention was first directed to exploring the electrochemical detection of l-ascorbic acid (vitamin C) which has been reported previously at nano-CoPC modified glassy carbon electrodes [19]. l-Ascorbic acid is a naturally occurring molecule which plays a vital part within mammalian metabolism as an antioxidant [30,31]. The detection of l-ascorbic acid is vital for medical diagnosis of scurvy [4,30–36]. We first consider the modification of a standard-SPE with nano-CoTAPC (see Experimental section) and explore this nano-CoTAPC modified SPE towards the sensing of l-ascorbic acid. Figure 3A shows a typical cyclic voltammetric profile where two voltammetric peaks are observed at ∼ +0.30 V (*vs.* SCE) and +0.90 V (*vs.* SCE). Note the former is not evident in the absence of l-ascorbic acid suggesting that this new peak is due to the electrocatalysis of CoPC. Figure 3B depicts the effect of increasing amounts (mass immobilised on the supporting electrode surface) of nano-CoTAPC upon the observed voltammetric peak height occurring at ∼ +0.30 V (*vs.* SCE) towards the electrochemical detection of l-ascorbic acid which shows that the peak current on the initial modification of the standard-SPE (5 μg) decreases as the amount of nano-CoTAPC is increased, suggesting that the underlying electrode becomes blocked by the CoPC nanoparticles. The peak observed at ∼ +0.90 V (*vs.* SCE) was monitored as a function of immobilised nano-CoTAPC and found to increase confirming that this peak is due to the Co^2+/3+^ electrochemical process which is in agreement with independent literature reports [5,13]. It is noted at this point, that multiple oxidative scans of the nano-CoTAPC SPEs result in a reduced signal meaning that a *new* electrode needs to be constructed each time. Hence the nano-CoTAPC takes the form of water insoluble micro-crystalline structures (see Figure 1C) which are then electrochemically oxidised from Co^2+^PC to Co^3+^PC undergoing solubilisation with the loss of material from the electrode surface and hence a loss/reduction in the voltammetric signal. Such a response has independently been reported for CoPC modified carbon electrodes [5].

If we consider further the origin of the electrocatalytic peak (∼ +0.30 V *vs.* SCE) observed in Figure 3A, a control experiment reveals that the direct electrochemical oxidation of L-ascorbic acid can be readily observed at the bare underlying/supporting electrode, as shown in Figure S3 which demonstrates that the electrochemical oxidation of l-ascorbic acid can be observed at bare standard-SPEs, GCE and BDDE at the potentials ∼ +0.30 V (*vs.* SCE), ∼ +0.80 V (*vs.* SCE) and ∼ +0.90 V (*vs.* SCE) respectively. Thus it is surmised that the peak observed in Figure 3A at ∼ +0.30 V (*vs.* SCE) is the response of the bare underlying electrode, with the peak at ∼ + 1.00 V (*vs.* SCE) being that of the Co^2+/3+^ couple. Modifications of the GCE and BDDE using nano-CoTAPC were next scrutinised towards the detection of l-ascorbic acid. Figure S4 shows voltammetric data where peaks are witnessed at ∼+1.10 V (*vs.* SCE) and ∼+0.90 V (*vs.* SCE) for GCE and BDDE respectively. It can be readily seen that through comparison of the voltammetric response in the absence and presence of the target analyte that the electrochemical oxidation of the nano-CoTAPC overlaps with that of the direct electrochemical oxidation of the target analyte at the bare underlying electrode surface; consequently as the mass of the nano-CoTAPC is increased upon the electrode surface, the peak heights (as seen in Figure S4B,D) are observed to increase giving the false impression of electrocatalysis. In summary, no electrocatalysis is being observed using the nano-CoTAPC modified SPEs and a bare SPE electrode can give rise to a voltammetric signal at a lower overpotential. Analysis of CoPC was next scrutinised to see if such commercially available CoPC displays electrocatalytic properties toward l-ascorbic acid. Presented in Figure S5 is a coverage study of CoPC upon a standard-SPE, as previously witnessed, a peak at ∼+0.4 V is obtained for the bare standard-SPE. Analysis of the cyclic voltammetry shows that there is a detrimental effect upon the peak height when increasing coverages of 5 μg to 70 μg are immobilised onto a standard-SPE.

Consideration was next turned towards the use of the bulk-CoPC SPEs which have recently been explored towards the sensing l-ascorbic acid [37]. A scan rate study was carried out towards 1 mM l-ascorbic acid in a pH 7.4 PBS using bulk-CoPC SPEs using a new electrode after each scan rate due to forming the water soluble Co^3+^PC upon the anodic scan. Figure S6 depicts typical cyclic voltammograms recorded over the range of 5 to 500 mVs^−1^ which show characteristic oxidation peaks for the direct electrochemical oxidation of l-ascorbic acid. In comparison with that of Figure S2, the first voltammetric peak at ∼ +0.20 V (*vs.* SCE) can be assigned to the direct electrochemical oxidation of l-ascorbic acid at bare (unmodified) SPE electrode surfaces, with the second peak arising from the Co^2+/3+^ couple. Analysis of the voltammetric peaks in the form of a plot of peak height *vs.* the square root of scan rate were found to exhibit a linear relationship over the experimentally chosen scan rate range indicating a diffusional process is in operation (bulk-CoPC SPE: *I_p_*/μA = 2.10 μA/(V s^−1^)^1/2^ + 2.48 μA, *R*^2^ = 0.99, *N* = 10). In fact, a similar response is observed if a bare SPE is used (see Figure S1) and analysis of the voltammetric peak height as a function of square root of scan rate reveals a linear response with a similar gradient (standard-SPE: *I_p_*/μA = 1.44 μA/(V s^−1^)^1/2^ + 0.97 μA, *R*^2^ = 0.99, *N* = 10) similar to that observed above. It is clear that the direct electrochemical oxidation of l-ascorbic acid is possible and no real electrocatalysis using the CoPC incorporated into the bulk of the SPE is observed.

**Figure 4.**
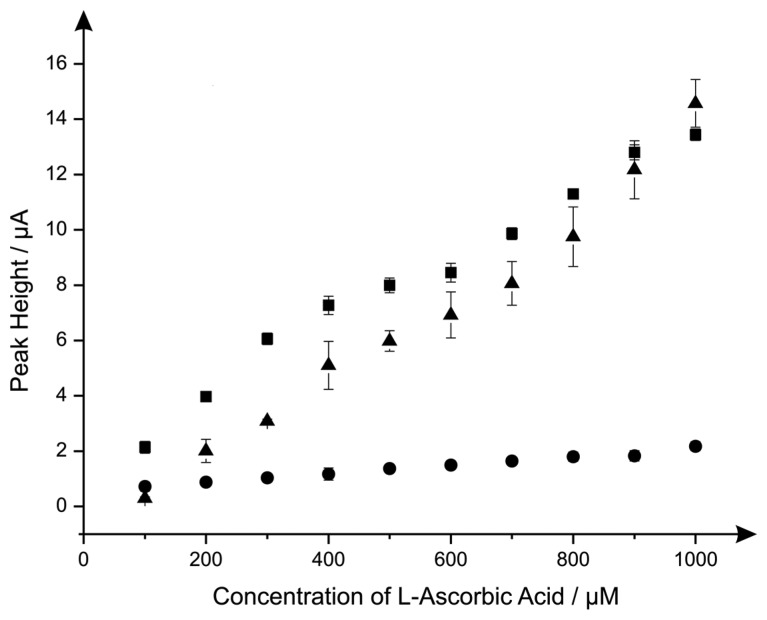
Calibration plots with error bars (*N* = 3) arising from the electroanalytical detection l-ascorbic acid over the concentration range 100 to 1000 μM within a pH 7.4 PBS using 20 μg nano-CoTAPC SPEs (circles), CoPC-SPEs (squares) and a standard-SPE (triangles). The voltammetric peak observed at ∼ +0.30 V (*vs.* SCE) was utilised to construct the calibration plot, with a new electrode used at each concentration. Scan rate: 100 mV s^−1^ using cyclic voltammetry.

Last the electroanalytical sensing of l-ascorbic acid using the CoPC modified electrodes was explored with additions made over the concentration range of 100 μM to 1000 μM. Figure 4 shows a comparison of the responses of the standard-SPE, nano-CoTAPC and the bulk-CoPC SPEs where it is clear that the nano-CoTAPC SPEs exhibit two linear ranges; 100 to 600 μM and 600 to 1000 μM (nano-CoTAPC SPE, lower range: *I_p_*/μA = 1.58 μA μM^−1^ + 0.56 μM, *R^2^* = 0.99, *N* = 5, higher range: *I_p_*/μA = 1.55 μA μM^−1^ + 0.55 μM, *R^2^* = 0.92, *N* = 5. CoPC SPE: *I_p_*/μA = 1.29 × 10 μA mM^−1^ + 1.81 mM, *R^2^* = 0.97, *N* = 10) and one linear range for the standard-SPE (*I_p_*/μA = 1.47 × 10 μA mM^−1^ − 1.29 mM^−1^, *R^2^* = 0.98, *N* = 10).

The bulk-CoPC SPEs and standard-SPEs show exceptional yet similar electrochemical performances over to that of the nano-CoTAPC SPE since the bare underlying electrode is the origin of the electroanalytical signal. In the case of the nano-CoTAPC, this configuration has the greatest coverage of CoPC and hence the least unmodified carbon surface therefore the underlying electrode is “locked” and therefore the signal is reduced as the CoPC has become saturated on the surface. Of course this coverage can be reduced but the reason to do this is meaningless as the bare unmodified SPE is the origin of the electroanalytically useful response. This observation that the electrocatalytic oxidation of l-ascorbic acid by CoPC [19] can be reproduced on the bare underlying electrode is reported for the first time; we note that Kozub *et al.* [5] reported similarly the electrocatalytic oxidation of nitrite by CoPC could be electroanalytical detected on bare carbon electrodes in the absence of CoPC.

**Figure 5.**
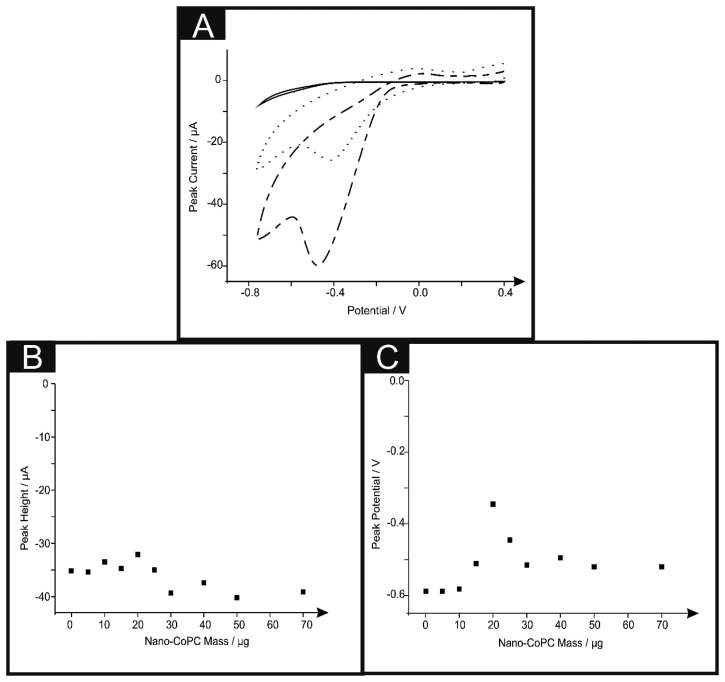
Cyclic voltammograms (**A**) recorded in an nitrogen degassed (solid line) and an oxygen saturated 0.1 M H_2_SO_4_ solution utilising a standard-SPE (short dashed line), bulk-CoPC SPE (dotted line) and a standard-SPE modified with 20 μg nano-CoTAPC (dotted-dashed line). Corresponding plots of coverage of peak height *vs.* nano-CoTAPC mass (**B**) and peak potential (**C**). Scan rate: 100 mVs^-1^

We next turned our attention to the possible catalytic effect of the CoPC SPEs towards the electrochemical reduction of oxygen which has been reported to be electrocatalytic with CoPC [13,28,38–42]. Figure 5A shows typical cyclic voltammograms for the electrochemical reduction of oxygen, where clear reduction peaks are evident using a nano-CoTAPC SPE ∼ −0.45 V (*vs.* SCE), bulk-CoPC SPE ∼ −0.40 V (*vs.* SCE) and a bare standard SPE ∼ −0.50 V (*vs.* SCE) which are in good agreement with previous literature concerning the reduction of oxygen. It is apparent upon inspection of this data that there is an increase in peak height and a shift to less negative potentials [13,38,39,41]. Figure 5B illustrates a coverage study upon a standard-SPE, where it is clear that above 30 μg of nano-CoTAPC modified upon the surface of the standard-SPE, saturation is reached and therefore magnitude of the peak height starts to plateau off. The observed response of peak potential as a function of coverage can be inferred from the work of Ward *et al.* [43] where in the limit of irreversible kinetics, as is the case here, an increase in the nano-CoTAPC coverage effects the peak potential. Quantitatively for hemi-spherical particles, in this case, nano-CoTAPC sitting upon the SPE, the peak potential (*Ep*) shifts quantitatively as given by [43]:

(2) $$E_{p}=E_{f}-\frac{RT}{\alpha F}\left[0.780-\ln(2\varphi k^{0})+\ln\left(\sqrt{\frac{\alpha \text{FDv}}{RT}}\right)\right]$$

where, *E_f_,* is the formal potential, *R* and *F* hold their standard values, *T*, is 298 K, *α*, is 0.5, *D*, is the diffusion coefficient for the analyte of choice, *k^0^*, is the heterogeneous rate constant and *υ*, is the scan rate. The surface area ratio, *φ*, is defined as:

$$\varphi =\frac{\text{surface area of nano}-\text{CoTAPC particle}}{\text{geometric area of the SPE}}$$

In our case the response observed in Figure 5C is consistent with Equation (2) where at low nano-CoTAPC coverages the voltammetric peak potential shifts from a high, (∼−0.80 V) overpotential to a lower (∼−0.40 V) value as the coverage increases. As theoretical simulations have shown, in addition to the shift in peak potential this is accompanied by an increase in peak height.

The effect of scan rate upon the voltammetric signal was next performed using each of the electrodes utilised throughout this work. Analysis in the form of plots of peak height *vs.* square root of the scan rate were analysed finding a linear response indicating a diffusional process is in operation (Standard-SPE: *I_p_*/μA = −2.22 μA/(V s^−1^)^1/2^ – 5.93 μA, *R*^2^ = 0.96, *N* = 10. Nano-CoTAPC SPE: *I_p_*/μA = −4.77 μA/(V s^−1^)^1/2^ + 2.87 μA, *R*^2^ = 0.98, *N* = 10. Bulk-CoPC SPE: *I_p_*/μA = 5.07 μA/(V s^−1^)^1/2^ + 11.1 μA, *R*^2^ = 0.99, *N* = 10). To further comprehend the electroanalytical signatures as shown in Figure 5A, Tafel analysis was performed which involves the analysis of the voltammograms corresponding to the electrochemical reduction of oxygen:

(3) $$a_{c}=-(RT/nF)(d\ln|j_{c}|dE$$

The cathodic transfer coefficient (*α*_c_) can be deduced from the slope of the plot of the cathodic current density (ln|*j_c_*|) against the applied potential (*E*). The cathodic Tafel slope is therefore referred to as the derivative d*E*/dln|*j*_c_|. It is implied that ln|*j_c_*| has a dimension of 1 [44]. Using Equation (3), Tafel analysis revealed a gradient of 315 mV, 134 mV and 135 mV for the bulk-CoPC SPEs, nano-CoTAPC SPE and bare SPEs respectively. We note that independent Tafel analysis reported a value of 209 mV for CoPC [45]. From these experimentally deduced Tafel values, (*α_c_*) values of 0.19, 0.44 and 0.44 are evident for the bulk-CoPC SPEs, nano-CoTAPC SPE and bare SPEs respectively suggesting that the transfer of the first electron is electrochemically irreversible in all cases. The electrochemical reduction on carbon electrodes goes through a 2 electron process to form the undesirable hydrogen peroxide [46] while metal phthalocyanines have been reported to undergo a CE process^45^: Co(II)PC + O_2_ ➔ Co(III)O_2_^−^; Co(III)O_2_^−^ + e^−^ ➔ product + Co(III)PC; Co(III) + e^−^ ➔ Co(II) with recent work indicating that CoPC produces both the undesirable and desirable products H_2_O_2_ and H_2_O [41]. Thus two differing electrochemical mechanisms are likely in operation, it is apparent that CoPC modifications provide no substantial electrocatalytic effects, as evidenced from the observation of peak potential over that of using a bare SPE. A difference however is observed from the magnitude of the voltammetric peak heights where the nano-CoTAPC gives rise to the largest improvement, due to the significant increase in surface area.

Next attention was turned to the electroanalytical sensing of hydrazine, a compound which has a vast usage in fields of rocket fuels, missile systems, weapons of mass destruction, fuel cells and corrosive inhibitors [39,41,47]. However there have been extensive reports focusing on its detrimental effects upon the human body, such as: irritation of the eyes, nose, and throat, dizziness, headache, nausea, pulmonary oedema, seizures, and coma in humans [48–50]. Hydrazine has also been reported to be readily oxidised by peroxidases, creating reactive intermediates within the human body which can cause severe side effects such as DNA manipulation, therefore demonstrating the carcinogenic nature of hydrazine [47].

**Figure 6.**
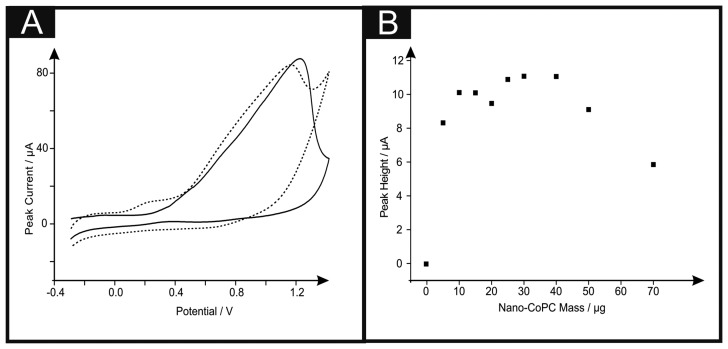
Cyclic voltammograms (**A**) utilising a standard-SPE (solid line), a 20 μg nano-CoTAPC SPE in the presence (dotted line) and absence (dashed line) of 500 μM Hydrazine in pH 7.4 PBS. Corresponding plots of nano-CoTAPC mass *vs.* peak height (**B**). Scan rate: 100 mV s^−1^.

Figure 6A shows the cyclic voltammetric response of hydrazine utilising a nano-CoTAPC SPE. It is apparent that the standard-SPE gives no distinctive oxidation peak towards this analyte however upon CoPC modification, a large oxidation peak at ∼+0.90 V (*vs.* SCE) in the absence of hydrazine is observed, confirming that this is the response for the Co^2+/3+^ couple, also shown is the response in the presence of hydrazine ∼+0.30 V (*vs.* SCE), thus confirming that the nano-CoTAPC acts as an electrocatalyst, towards hydrazine; such findings are in agreement with previous literature, utilising bulk-CoPC-SPEs [26]. Figure 6B depicts a plot of peak height as a function of the mass of nano-CoTAPC modified upon the surface of the standard-SPE respectively, upon excessive modification (>30 μg) of the working electrode causes reduced electrochemical performance, proven with a decrease of peak height.

Dilution of the nano-CoTAPC was next explored *via* the same study where it was visible that a catalysed peak is still present even at a masses of as small as 5.00 × 10^−4^ μg. The bulk-CoPC SPEs show similar responses (shown in Figure S7) to that of our previously reported literature, when utilising this type of CoPC SPE towards hydrazine, with a significant oxidation peak at ∼+0.50 V (*vs.* SCE).

**Figure 7.**
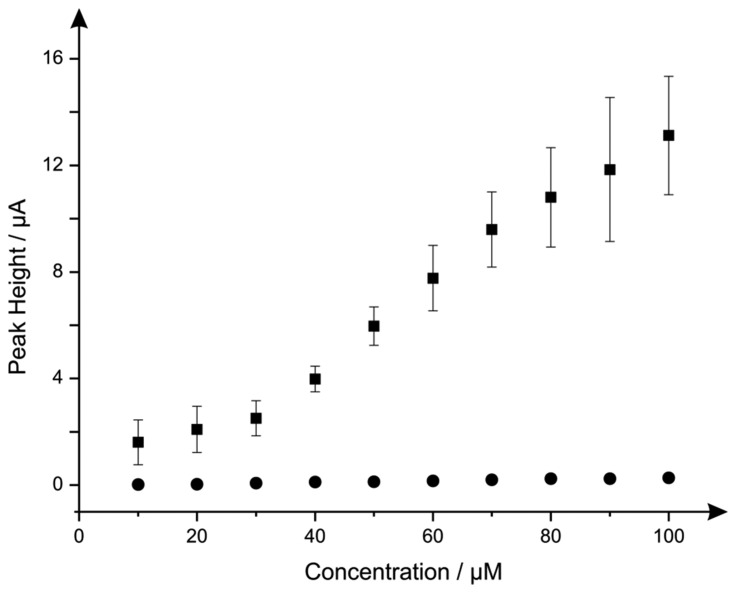
Calibration plots showing nano-CoTAPC SPE (squares) and CoPC-SPE (circles) towards additions of (10 μM to 100 μM) hydrazine into pH 7.4 PBS, utilising a *new electrode* after each addition. Scan rate: 100 mV s^−1^. In both cases error bars are presented for nano-CoTAPC SPE (squares) and CoPC-SPE (circles) responses where the average of three measurements and standard-deviations are presented.

Additions of hydrazine into a pH 7.4 PBS were next explored using the nano-CoTAPC SPEs and bulk-CoPC SPEs. Figure 7 illustrates the electroanalytical detection of hydrazine, for both SPEs over concentration range of 10 μM to 100 μM (nano-CoTAPC SPE: *I_p_*/μA = 3.00 × 10^−3^ μA μM^−1^ − 0.79 μM, *R^2^* = 0.98, *N* = 9; bulk-CoPC SPE: *I_p_*/μA = 3.00 × 10^−3^ μA μM^−1^ − 0.40 μM, *R^2^* = 0.98, *N* = 10). Upon inspection of the analytical data for nano-CoTAPC SPE, two linear ranges were determined with the first linear range (10 to 30 μM) giving rise to a limit of detection (*3σ* ) of 9.00 μM compared to that of the bulk-CoPC SPEs with a value corresponding to 6.21 μM, which is better than CoPC polymeric-modified electrodes [14] and similar to other related cobalt phthalocyanine structures [51] However, the error bars presented within Figure 7, shows that the detection below 30 μM is less reproducible as bulk-CoPC SPEs.

In summary this report has shown the true “electrocatalysis” of hydrazine utilising both CoPC electrode configurations, it is also apparent that the drop casted nano-CoTAPC can lead to an improved peak height, for the reduction of oxygen compared to that of a bulk-CoPC SPE and the standard-SPE. Finally, it has been made clear that l-ascorbic acid has no electrocatalytic activity when utilised with a CoPC catalyst via either method, and that useful electrochemical analysis can be made using a bare underlying electrode.

## 4. Conclusions

CoPC modified electrodes prepared by drop casting CoPC nanoparticles onto a range of carbon- based electrode substrates are critically compared with CoPC bulk modified screen-printed electrodes in the sensing of the model analytes l-ascorbic acid, oxygen and hydrazine. Coverage analysis of both CoPC configurations found that the drop casted nano-CoTAPC showed 10 times less than the literature value obtained from the charge of the system for a CoPC monolayer, however SEM analysis and coverage values suggest microcrystalline structures therefore indicating that the immobilised CoPC on the surface are generally nonconductive and the residing edge plane sites/defects on the underlying electrode are the electrochemically active regions; however the amount of CoPC upon the surface can easily be changed to optimise the detection of the model analyte under investigation. In terms of the bulk modified CoPC, only the surface layer will be accessible to the solution and hence the rest of the electrode containing the bulk of CoPC is “dead space” as the electrocatalytic species cannot access this (lack of a triple phase boundary). As these electrodes are screen-printed using a mediated CoPC ink the amount of CoPC cannot be easily changed, however such techniques produce highly reproducible economical disposable one-shot sensors.

We have reported that that no electrocatalysis occurs at both types of CoPC electrode configurations towards the detection of l-ascorbic acid, and it is clear that the bare underlying electrode provides suitable voltammetric signals for the analytical detection. It seems that such realisation provides new insights into previous literature reports suggesting “electrocatalysis”, without any clear control experiments. The electrochemical reduction of oxygen in acidic medium has been reported showing minor electrocatalysis, however an improvement in peak height is witnessed due to a larger CoPC surface area upon the electrode surface. On the other hand true electrocatalysis is observed toward hydrazine where no such voltammetric features are witnessed on the bare underlying electrode substrate.

The clear advantage of the nano-CoPC is that differing coverages can be utilised and tailored to provide optimal analytical signals but can also givevariable reproducibility. In the bulk-CoPC SPE's, due to their fabrication approach the amount of electrocatalyst cannot be easily altered but give rise to very reproducible voltammetric signals; these observations are succinctly summarised in Figure 7 towards to electroanalytical sensing of hydrazine.

## References

[1] Zinola C.F., Martins M.E., Tejera E.P., Neves N.P. (2012). Electrocatalysis: Fundamentals and Applications. Int. J. Electrochem..

[2] Preidel W., Saegar S., von Lucadou I., Lager W. (1991). An electrocatalytic glucose sensor for *in-vivo* application. Biomed. Instrum. Technol..

[3] Morozan A., Jousselme B., Palacin S. (2011). Low-platinum and platinum-free catalysts for the oxygen reduction reaction at fuel cell cathodes. Energy Environ. Sci..

[4] Zuo X., Zhang H., Li N. (2012). An electrochemical biosensor for determination of ascorbic acid by cobalt (II) phthalocyanine-multi-walled carbon nanotubes modified glassy carbon electrode. Sens. Actuators B Chem..

[5] Kozub B.R., Compton R.G. (2010). Voltammetric studies of the redox mediator, cobalt phthalocyanine, with regard to its claimed electrocatalytic properties. Sens. Actuators B Chem..

[6] Gilmartin M.A.T., Hart J.P., Patton D.T. (1995). Prototype, solid-phase, glucose biosensor. Analyst.

[7] Santos L.M., Baldwin R.P. (1987). Liquid chromatography/electrochemical detection of carbohydrates at a cobalt phthalocyanine containing chemically modified electrode. Anal. Chem..

[8] Mashazi P.N., Ozoemena K.I., Nyokong T. (2006). Tetracarboxylic acid cobalt phthalocyanine SAM on gold: Potential applications as amperometric sensor for H_2_O_2_ and fabrication of glucose biosensor. Electrochim. Acta.

[9] Ozoemena K.I., Nyokong T. (2006). Novel amperometric glucose biosensor based on an ether-linked cobalt(II) phthalocyanine-cobalt(II)tetraphenylporphyrin pentamer as a redox mediator. Electrochim. Acta.

[10] Kondo T., Horitani M., Yuasa M. (2012). Sensitive Electrochemical Detection of Glucose at Glucose Oxidase-Cobalt Phthalocyanine-Modified Boron-Doped Diamond Electrode. Int. J. Electrochem..

[11] Wring S.A., Bracey L., Birch B.J., Hart J.P. (1990). Development of screen-printed carbon electrodes, chemically modified with cobalt phthalocyanine, for electrochemical sensor application. Anal. Chim. Acta..

[12] Korfhage K.M., Ravichandran K., Balwin R.P. (1984). Phthalocyanine-containing chemically modified electrodes for electrochemical detection in liquid chromatography/flow injection systems. Anal. Chem..

[13] Issacs M., Aguirre M.J., Toro-Labbe A., Costamanga J., Paez M., Zagal J.H. (1998). Comparative study of the electrocatalytic activity of cobalt phthalocyanine and cobalt naphthalocyanine for the reduction of oxygen and the oxidation of hydrazine. Electrochim. Acta.

[14] Peng Q., Guarr T.F. (1994). Electro-oxidation of hydrazine at electrodes modified with polymeric cobalt phthalocyanine. Electrochim. Acta.

[15] Geraldo D.A., Tongo C.A., Limson J., Nyokong T. (2008). Electrooxidation of hydrazine catalyzed by noncovalently functionalized single-walled carbon nanotubes with CoPc. Electrochim. Acta.

[16] Chicharro M., Zapardiel A., Bermejo E., Madrid E., Rodriguez C. (2002). Flow Injection Analysis of Aziprotryne Using an Electrochemical Sensor Based on Cobalt Phthalocyanine Modified Carbon Paste Electrode. Electroanalysis.

[17] Caro C.A., Bedioui F., Zagal J.H. (2002). Electrocatalytic oxidation of nitrite on a vitreous carbon electrode modified with cobalt phthalocyanine. Electrochim. Acta.

[18] Matemadombo F., Apetrei C., Nyokong T., Rodríguez-Méndez M.L.R., de Saja J.A. (2012). Comparison of carbon screen-printed and disk electrodes in the detection of antioxidants using CoPc derivatives. Sens. Actuators B Chem..

[19] Wang K., Xu J., Tang K., Chen H. (2005). Solid-contact potentiometric sensor for ascorbic acid based on cobalt phthalocyanine nanoparticles as ionophore. Talanta.

[20] Agboola B.O., Mocheko A., Pillay J., Ozoemena K.I. (2008). Nanostructured cobalt phthalocyanine single-walled carbon nanotube platform: Electron transport and electrocatalytic activity on epinephrine. J. Porphyr. Phthalocyanines.

[21] Pillay J., Vilakazi S. (2012). Nanostructured metallophthalocyanine complexes: Synthesis and electrocatalysis. J. Porphyr. Phthalocyanines.

[22] Welch C.M., Compton R.G. (2006). The use of nanoparticles in electroanalysis: A review. Anal. Bioanal. Chem..

[23] Patoisa T., Sanchez J., Berger F., Fievet P., Segut O., Moutarlier V., Bouvet M., Lakard B. (2013). Elaboration of ammonia gas sensors based on electrodeposited polypyrrole—Cobalt phthalocyanine hybrid films. Talanta.

[24] Sun X., Li F., Shen G., Huang J., Wang X. (2014). Aptasensor based on the synergistic contributions of chitosan-gold nanoparticles, graphene-gold nanoparticles and multi-walled carbon nanotubes-cobalt phthalocyanine nanocomposites for kanamycin detection. Analyst.

[25] Hosseini H., Mahyari M., Bagheri A., Shaabani A. (2014). A novel bioelectrochemical sensing platform based on covalently attachment of cobalt phthalocyanine to graphene oxide. Biosens. Bioelectron..

[26] Foster C.W., Metters J.P., Kampouris D.K., Banks C.E. (2014). Ultraflexible Screen-Printed Graphitic Electroanalytical Sensing Platforms. Electroanalysis.

[27] Honeychurch K.C., Gilbert L., Hart J.P. (2010). Electrocatalytic Behaviour of Citric Acid at a Cobalt Phthalocyanine Modified Screen-printed Carbon Electrode and Its Application in Pharmaceutical and Food Analysis. Anal. Bioanal. Chem..

[28] Yang G., Wang K., Xu J., Chen H. (2005). Determination of Theophylline in Drugs and Tea on Nanosized Cobalt Phthalocyanine Particles Modified Carbon Paste Electrode. Anal. Lett..

[29] Pereira-Rodrigues N., Cofr R., Zagal J.H., Bedioui F. (2007). Electrocatalytic activity of cobalt phthalocyanine CoPc adsorbed on a graphite electrode for the oxidation of reduced l-glutathione (GSH) and the reduction of its disulfide (GSSG) at physiological pH. Bioelectrochemistry.

[30] Traber M.G., Stevens J.F. (2011). Vitamins C and E: beneficial effects from a mechanistic perspective. Free Radic. Biol. Med..

[31] Davey M.W., Montagu M.V., Inzé D., Sanmartin M., Kanellis A., Smirnoff N., Benzie I.J.J., Strain J.J., Favell D., Fletcher J. (2002). Plant l-ascorbic acid: chemistry, function, metabolism, bioavailability and effects of processing. Sci. Food Agric..

[32] Rinehart J.F., Greenberg L.D. (1942). The detection of subclinical scurvy or vitamin C deficiency. Ann. Intern. Med..

[33] Zhuang Z., Li J., Xu R., Xiao D. (2011). Electrochemical Detection of Dopamine in the Presence of Ascorbic Acid Using Overoxidized Polypyrrole/Graphene Modified Electrodes. Int. J. Electrochem. Sci..

[34] Ma X., Chao M., Wang Z. (2012). Electrochemical detection of dopamine in the presence of epinephrine, uric acid and ascorbic acid using a graphene-modified electrode. Anal. Methods.

[35] Wring S.A., Hart J.P. (1990). Voltammetric behaviour of ascorbic acid at a graphite—epoxy composite electrode chemically modified with cobalt phthalocyanine and its amperometric determination in multivitamin preparations. Anal. Chim. Acta.

[36] Li J., Hu M., Yu R. (1996). Pressed-pellet solid potentiometric sensor for ascorbic acid based on derivatives of cobalt(II) phthalocyanine doped with iodine. Sens. Actuators B Chem..

[37] Chen P.Y., Luo C.H., Chen M.C., Tsai F.J., Chang N.F., Shih Y. (2011). Screen-printed carbon electrodes modified with cobalt phthalocyanine for selective sulfur detection in cosmetic products. Int. J. Mol. Sci..

[38] Elzing A., van der Putten A., Visscher W., Barendrecht E. (1986). The cathodic reduction of oxygen at cobalt phthalocyanine: Influence of electrode preparation on electrocatalysis. J. Electroanal. Chem. Interfacial Electrochem..

[39] Zelnick S.D., Mattie D.R., Stepaniak P.C. (2003). Occupational Exposure to Hydrazines: Treatment of Acute Central Nervous System Toxicity. Aviat. Space Envir. Med..

[40] Kobayashi P.J.N., Leveres A.B.P. (1992). Cathodic reduction of oxygen and hydrogen peroxide at cobalt and iron crowned phthalocyanines adsorbed on highly oriented pyrolytic graphite electrodes. Inorg. Chem..

[41] Kruusenbergm L.M.I., Tammeveski K. (2013). Oxygen Electroreduction on Multi-Walled Carbon Nanotube Supported Metal Phthalocyanines and Porphyrins in Acid Media. Int. J. Electrochem. Sci..

[42] El Hourch A., Belcadi S. (1992). Electrocatalytic reduction of oxygen at iron phthalocyanine modified polymer electrodes. J. Electrochem..

[43] Ward K.R., Gara M., Lawrence N.S., Hartshorne R.S., Compton R.G. (2013). Nanoparticle modified electrodes can show an apparent increase in electrode kinetics due solely to altered surface geometry: The effective electrochemical rate constant for non-flat and non-uniform electrode surfaces. J. Electroanal. Chem..

[44] Guidelli R., Compton R.G., Feliu J.M., Gileadi E., Lipkowski J., Schmickler W., Trasatti S. (2014). Defining the transfer coefficient in electrochemistry: An assessment (IUPAC Technical Report). Pure Appl. Chem..

[45] Yu E.H., Cheng S., Logan B.E., Scott K. (2009). Electrochemical reduction of oxygen with iron phthalocyanine in neutral media. J. Appl. Electrochem..

[46] Randviir E.P., Banks C.E. (2014). The Oxygen Reduction Reaction at Graphene Modified Electrodes. Electroanalysis.

[47] Mo J.W., Ogorevc B., Zhang X., Pihlar B. (2000). Cobalt and Copper Hexacyanoferrate Modified Carbon Fiber Microelectrode as an All-Solid Potentiometric Microsensor for Hydrazine. Electroanalysis.

[48] Nasirizadeh N., Zare H.R., Fakhari A.R., Ahmar H., Ahmadzadeh M.R., Naeimi A. (2011). A study of the electrochemical behavior of an oxadiazole derivative electrodeposited on multi-wall carbon nanotube-modified electrode and its application as a hydrazine sensor. J. Solid State Electr..

[49] Ahmar H., Keshipour S., Hosseini H., Fakhari A.R., Shaabani A., Bagheri A. (2013). Electrocatalytic oxidation of hydrazine at glassy carbon electrode modified with ethylenediamine cellulose immobilized palladium nanoparticles. Electroanal. Chem..

[50] United States Environmental Protection Agency Hydrazine Information.

[51] Ozoemena K.I. (2006). Anodic Oxidation and Sensing of Hydrazine at a Glassy Carbon Electrode Modified with Cobalt (II) Phthalocyanine–cobalt (II) Tetraphenylporphyrin (CoPc-(CoTPP)4) Supramolecular Complex. Sensors.

